# Cardioneuroablation for successful treatment of symptomatic bradycardia in a 12-year-old child after a 6-month follow-up

**DOI:** 10.3389/fcvm.2023.1290482

**Published:** 2023-11-29

**Authors:** Xin Xu, Shuang He, Qian Liu, Ruixi Liu, Lei Zhang, Weijie Chen, Yuehui Yin, Tiewei Lu

**Affiliations:** ^1^ Department of Cardiology, Children’s Hospital of Chongqing Medical University, National Clinical Research Center for Child Health and Disorders, Ministry of Education Key Laboratory of Child Development and Disorders, Chongqing Key Laboratory of Pediatrics, Chongqing, China; ^2^ Department of Cardiology, The Second Affiliated Hospital of Chongqing Medical University, Chongqing, China

**Keywords:** cardioneuroablation (CNA), sinus bradycardia, sinus arrest, ganglionated plexus, vagal tone, children

## Abstract

**Background:**

Cardioneuroablation (CNA) is recognized as a promising therapeutic option for adults with severe symptomatic bradycardia caused by excessive vagal tone. However, no pediatric cases have been reported to date. Therefore, the aim of this study is to evaluate the feasibility and efficacy of CNA in children.

**Methods:**

A 12-year-old male patient was hospitalized with symptoms of fatigue, palpitations, and syncope for more than 2 months, and was definitively diagnosed with functional sinoatrial node dysfunction by using a 12-lead electrocardiogram, 24-h Holter monitoring, loading dose of atropine test (0.04 mg/kg), and treadmill exercise test. Simultaneously, whole-exome sequencing was performed on the child and his core family members. After completing the preoperative examination and signing the informed consent form, the child underwent CNA therapy.

**Results:**

First, the electroanatomic structures of both atria were mapped out by using the Carto 3 system, according to the protocol of purely anatomy-guided and local fractionated intracardiac electrogram–guided CNA methods. Then, the local fractionated intracardiac electrograms of each cardiac ganglionated plexus (GP), including the GP between the aortic root and the medial wall of the superior vena cava, the GP between the posterior wall of the coronary sinus ostium and the left atrium, the GP between the anterior antrum of the right superior pulmonary vein and the superior vena cava, the GP in the superolateral area around the root of the left superior pulmonary vein, the GP around the root of the right inferior pulmonary vein, and the GP around the root of the left inferior pulmonary vein, were used as targets for ablation at a power of 30 W with an ablation index of 350–400. At a 6-month follow-up, the child's heart rhythm saw a complete restoration to sinus rhythm and clinical symptoms disappeared.

**Conclusion:**

The first application of CNA in a child with symptomatic sinus bradycardia was achieved with better clinical outcomes. CNA can be carried out cautiously in children under suitable indications.

## Introduction

Severe bradycardia with different etiologies can lead to serious clinical manifestations such as fatigue, palpitations, syncope, and even sudden death. The traditional therapy for patients with severe bradycardia is cardiac pacemaker implantation. However, when these patients are adolescents, or much younger, the children or their guardians refuse pacemaker implantations because of concerns about long-term complications (1–3). In recent years, studies have reported that cardioneuroablation (CNA) has become a promising therapeutic strategy for bradycardia caused by excessive vagal tone, and has achieved good clinical outcomes in adults (4–6). However, so far no case of a pediatric patient receiving CNA therapy has been reported. This study aims to fill this gap by presenting the case of a child with sinus arrest, sinus bradycardia, and junctional escape rhythms and showing improvement in clinical outcomes with the relieving of symptoms and rhythm disorders using CNA therapy.

## Methods and materials

### Study population

**Figure 1:**
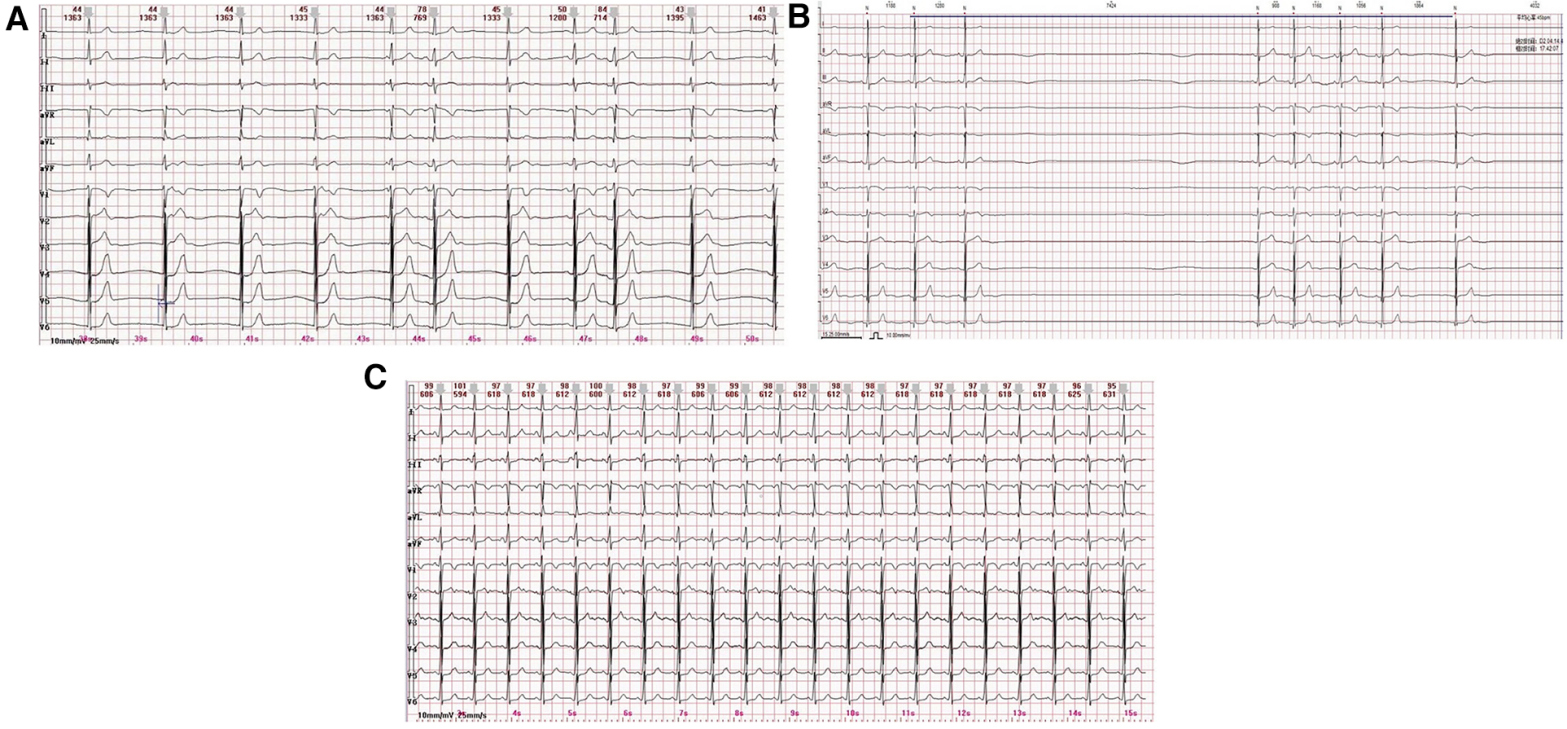
(**A**) 12-lead ECG displays a sinus bradycardia and junctional escape rhythm. (**B**) The 24 h Holter monitoring displays a sinus arrest of 7.424 s. (**C**) Normal sinus rhythm appears by a loading dose of atropine test (only the ECG graph after 1 min of atropine administration is displayed).

A 12-year-old male patient was hospitalized with symptoms of fatigue, palpitations, and syncope for more than 2 months. The 12-lead electrocardiogram (ECG) showed sinus bradycardia and junctional escape rhythm (Figure 1A). A 24 h Holter monitoring displayed a total of 62,108 heartbeats in 24 h, with 5,094 sinus arrests greater than 2 s and 971 sinus arrests greater than 3 s, with the longest cardiac arrest time being 7.424 s (Figure 1B). The child had no history of myocarditis prior to the onset of the disease, no structural cardiac abnormalities, no similar family history, and no positive findings on laboratory examinations. Normal sinus rhythm appeared after a loading dose of atropine test (0.04 mg/kg) and treadmill exercise test (Figure 1C). The child was diagnosed as having functional sinoatrial node dysfunction possibly due to excessive vagal tone based on the above results. Meanwhile, to exclude inherited heart disease, blood samples from the child, his elder sister, and his parents were collected, and whole-exome sequencing (WES) was performed by MyGenostics (Beijing, China) using the Illumina HiSeq X ten system. After strongly rejecting pacemaker implantation, the child's parents agreed to CNA therapy, for which they signed an informed consent form.

### CNA procedure

**Figure 2:**
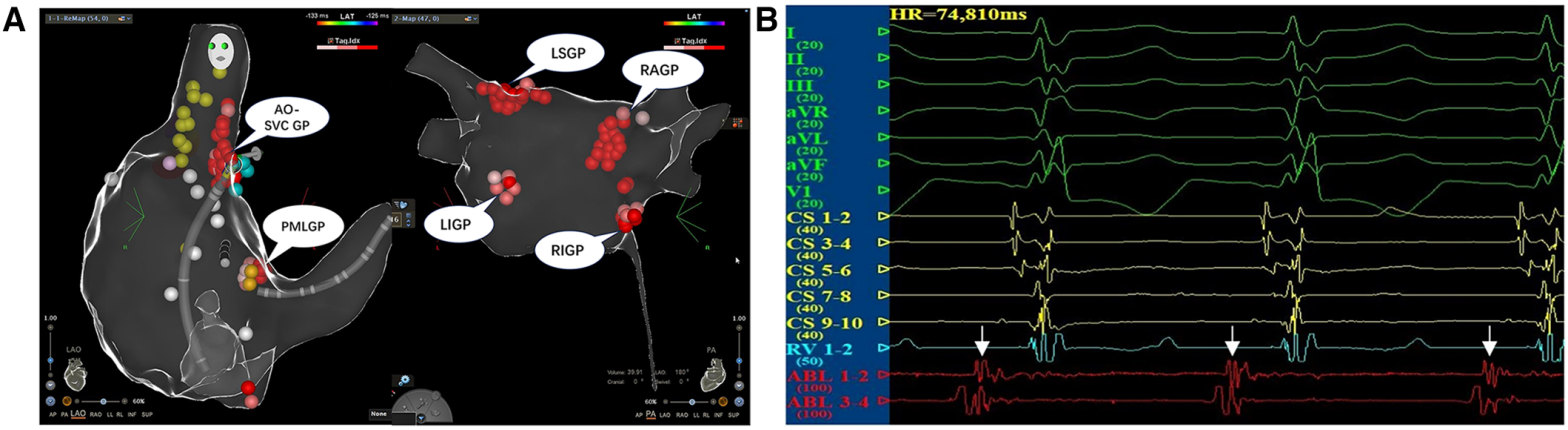
(**A**) The CNA procedure. The local fractionated intracardiac electrograms of each cardiac ganglionated plexus, including Ao-SVC GP, PMLGP, RAGP, LSGP, RIGP, and LIGP, were used as targets for ablation using a power of 30 W for 30 s with an ablation index of 350–400. (**B**) The local fractionated intracardiac electrograms of the ablation catheter from AO-SVC GP were used as the target potential during the CNA procedure.

After a satisfactory administration of anesthesia, the bilateral femoral veins were punctured successfully and a quadripolar electrode catheter was inserted into the right ventricle for an intracardiac electrophysiological study and right ventricle pacing. When severe bradycardia occurred in the patient during the procedure, a decapolar steerable electrode catheter was inserted into the coronary sinus for the intracardiac electrophysiological study. Surface ECG and intracardiac electrograms were continually monitored by using a multichannel recording system during the whole procedure (LEAD-7000; JJET, Chengdu, China). The three-dimensional (3D) electroanatomic structures of both atria were mapped out by using the Carto 3 system (Biosense Webster, Diamond Bar, CA, USA). The sinus atrial node, phrenic nerve, coronary sinus, and His bundle were labeled. A Thermocool® ST (Biosense Webster, Diamond Bar, CA, USA) irrigated catheter was inserted in the right/left atrium for the ablation therapy. According to the protocol reported by Pachon et al. and Aksu et al. (3, 7), we administered the CNA therapy by sequentially targeting the ganglionated plexus (GP) between the aortic root and the medial wall of the superior vena cava (Ao-SVC GP), the GP between the posterior wall of the coronary sinus ostium and the left atrium (PMLGP), the GP between the anterior antrum of the right superior pulmonary vein and the superior vena cava (RAGP), the GP in the superolateral area around the root of the left superior pulmonary vein (LSGP), the GP around the root of the right inferior pulmonary vein (RIGP), and the GP around the root of the left inferior pulmonary vein (LIGP) (Figure 2A). The local fractionated intracardiac electrograms (Figure 2B) of each cardiac GP, including Ao-SVC GP, PMLGP, RAGP, LSGP, RIGP, and LIGP, were used as ablation targets, and the radio frequency of the GPs was performed in the power-controlled mode in a point-by-point fashion through the irrigated ST catheter. Energy was delivered at a power of 30 W for 30 s with an ablation index of 350–400. The ablation endpoint was the complete elimination of local fractionated intracardiac electrograms in each GP. Low-molecular heparin was administrated intravenously at 100 IU/kg during the CNA procedure with an additional half-dose of over 2 h of operating time. The duration of radiofrequency ablation in the child was 3 h with a fluoroscopy time of 11 m and dose of 5.4 mGy. Aspirin antiplatelet aggregation was routinely administered for 6 months after the CNA procedure.

## Results

### Change in electrical characteristics after CNA

**Figure 3:**
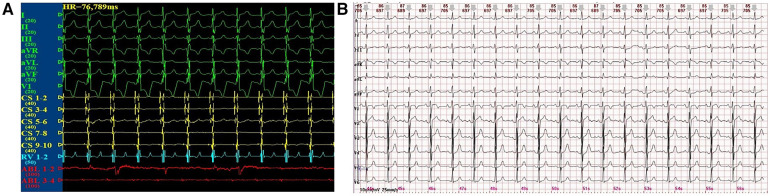
(**A**) Sinus rhythm after the CNA procedure. (**B**) 12-lead ECG at the 6-month follow-up after the CNA procedure.

After the completion of the purely anatomy-guided and local fractionated intracardiac electrogram–guided CNA procedure in the locations of the GPs, the child's surface ECG and intracardiac electrograms instantly shifted to sinus rhythm (Figure 3A). One week after the CNA procedure, the 12-lead ECG still presented sinus bradycardia and junctional escape rhythm, but the 24-h Holter monitoring displayed a total of 78,434 heartbeats in 24 h, with 143 sinus arrests greater than 2 s and one sinus arrest greater than 3 s, with the longest cardiac arrest time being 3.780 s (Figure 3B). Meanwhile, the child's palpitations were relieved significantly. At the 3-month follow-up, the child's 12-lead ECG completely restored to normal sinus rhythm, and 24 h Holter monitoring displayed a total of 120,457 heartbeats in 24 h without sinus arrest. At the 6-month follow-up, the child's 12-lead ECG still remained in normal sinus rhythm, and the 24 h Holter monitoring displayed a total of 102,912 heartbeats in 24 h without sinus arrest. Interestingly, the child's palpitations and syncope disappeared completely. A comparison of Holter features before and after the CNA procedure revealed a gradual increase in the total number of heartbeats, a marked improvement in cardiac rhythm, and a gradual disappearance of sinus arrests (Table 1). The deceleration capacity (DC) of the heart rate, a new technique for assessing autonomic tone, is used to quantitatively assess vagal tone by analyzing the overall trend of sinus rhythm over a 24 h period. Normally, DC is positively correlated with vagal tone, but in our patient, the DC was 14.4 before CNA treatment and only 8.3 after CNA treatment, which confirmed that the child's symptoms and rhythm disturbances were closely related to high vagal tone.

**Table 1:** Change of Holter feature before and after the CNA procedure.

	Total number of heartbeats in 24 h	Cardiac rhythm (sinus/junctional)	Number of cardiac arrests >2 s	Number of cardiac arrests >3 s
Before CNA	62,108	Less sinus/more junctional	5,094	971
1 week after CNA	78,434	Less sinus/more junctional	143	1
3 months after CNA	120,457	Sinus	0	0
6 months after CNA	102,912	Sinus	0	0

### WES outcome

**Figure 4:**
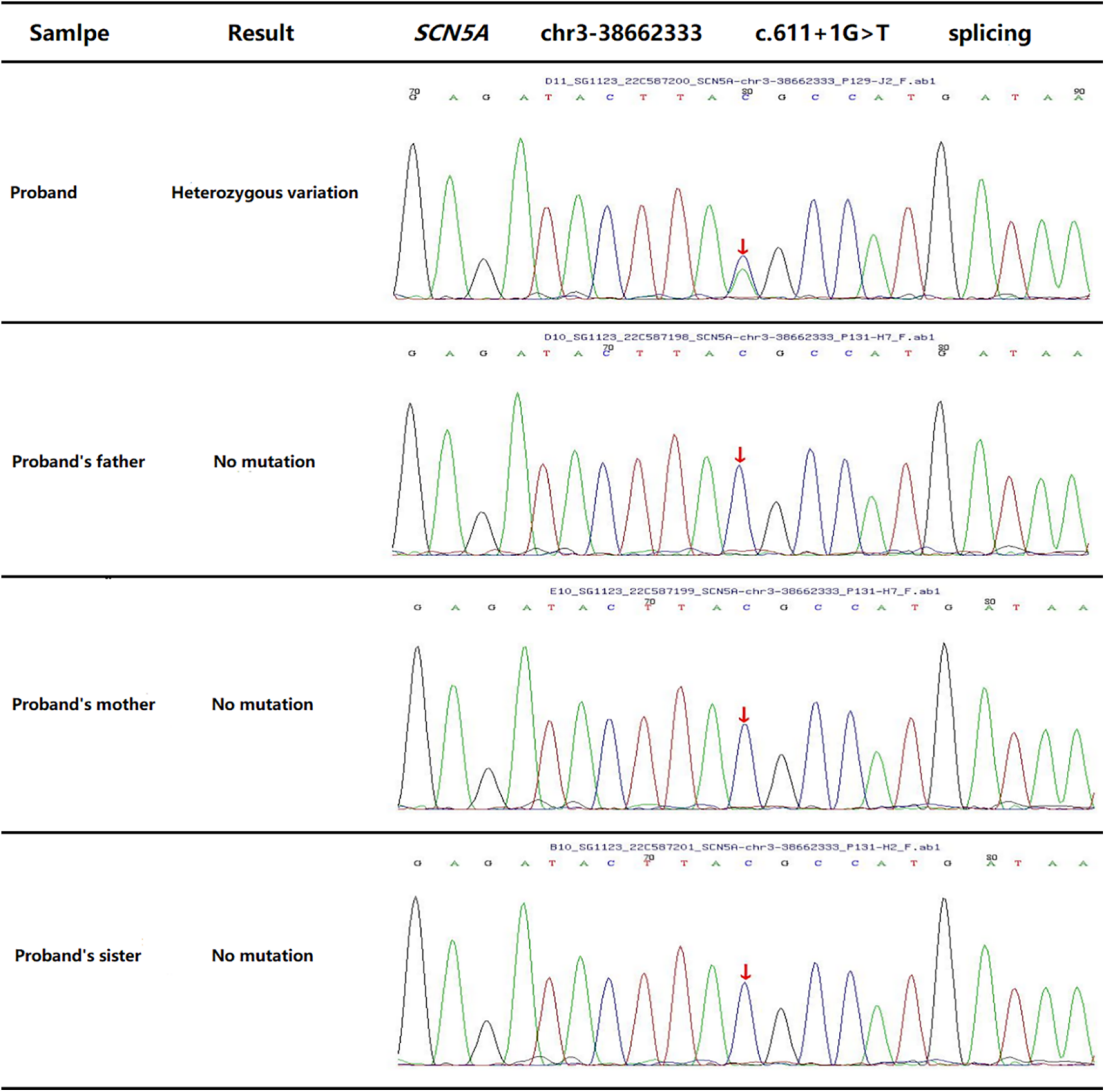
WES outcome of proband (the child) and his family members displays a novel c.611 + 1G>T heterozygous mutation in the *SCN5A* gene.

The child’s parents and sister had no previous history of similar illnesses. WES was performed on the child and his core family members. It showed the child had a novel c.611 + 1G>T heterozygous mutation in the *SCN5A* gene (Figure 4), which resulted in a splicing mutation of amino acids. No abnormalities were found in the other family members.

### Complication

No intraoperative or postoperative puncture/ablation-related complications occurred in the child, such as vascular injury, arrhythmia, laryngeal reentrant nerve injury, and pericardial tamponade. Heart structure and function remained normal during follow-up.

## Discussion

This study displayed the first application of CNA in a child with symptomatic bradycardia. The child's heart rhythm was completely restored to sinus rhythm and clinical symptoms disappeared without any perioperative and long-term complications during the 6-month follow-up. Overall, this preliminary result was inspiring, showing that CNA may be safe and feasible for children with symptomatic bradycardia.

### Principle of the CNA procedure for treating bradycardia

It is well-known that the autonomic nervous system plays an important role in cardiac functions. An imbalance in autonomic tone, mainly excessive vagal tone, is the common cause of functional sinoatrial node dysfunction (SND), functional atrioventricular block (AVB), and vasovagal syncope (VVS), except organic cardiac lesions (7–9). Traditionally, bradycardic patients who are at a high risk for clinically serious cardiovascular events if they developed hemodynamic disturbances may require pacemaker implantation to prevent sudden fatal events (1, 2). The cell bodies of cardiac efferent vagal postganglionic neurons have been shown to be contained in the intrinsic cardiac GP, which is embedded in the atrial wall and in epicardial fat pads (10, 11). Common GPs include Ao-SVC GP, PMLGP, RAGP, RIGP, LSGP, and LIGP with different physiological functions, and they interact with one another (6, 9, 10). Therefore, a disruption of nerve fibers by radiofrequency ablation can inhibit excessive activation of the vagal nerve, thereby eliminating or improving clinical symptoms. That is why CNA has proven to be a reliable therapeutic target for patients with excessive vagal tone.

In our study, our child patient presented with abnormal rhythm (sinus bradycardia, sinus arrest, and junctional escape rhythm) and symptoms of fatigue, palpitations, and syncope, while organic cardiac disorders and family history were excluded. Moreover, the resting heart rate of the child was approximately 58 bpm with sinus bradycardia and junctional escape rhythm, which rose to 97 bpm with sinus rhythm after atropine intravenous administration, which proved that the result of the atropine test was negative. Therefore, symptomatic bradycardia was considered to have been caused by excessive vagal tone. All these show that CNA is able to achieve better clinical outcomes.

### Suitable indications for CNA

Currently, there is a lack of guidelines for the selection of suitable candidates for CNA. Some authors have reported that indications for CNA in adults include the following: (1) patients with severe symptomatic bradycardia of functional SND or AVB [bradycardia was documented by performing ECG and 24 h Holter monitoring and was associated with excessive vagal tone with evidence of correcting the bradycardia by a loading dose of atropine], or patients with a symptomatic vasovagal syncope (cardioinhibitory type or mixed type), which was documented by performing a head-up tilt table test (HUT); (2) patients aged between 14 and 65 years; (3) those with no structural heart diseases; and (4) those who rejected the option of implanting a cardiac pacemaker on them (6). However, no indications were noted for CNA in children.

The child patient in our study had no structural heart diseases, except for a novel c.611 + 1G>T heterozygous mutation in the *SCN5A* gene. Many studies have reported that *SCN5A*, encoding the pore-forming ion-conducting *α*-subunit of the cardiac sodium channel (Nav1.5), was the causative gene for ion channel disease, including sick sinus syndrome, long Q-T syndrome, and Brugada syndrome (12–14). It is worth considering that the child had a heterozygous mutation in the *SCN5A* gene but a negative atropine test, along with a satisfactory clinical outcome and DC value reduction after CNA. However, further basic research is needed to clarify the relationship between the novel c.611 + 1G>T heterozygous mutation and disease development. Meanwhile, this study provides probable clinical evidence for the development of CNA indications in pediatric patients in the future.

### Therapeutic strategy of the CNA procedure

There is no guideline for the therapeutic strategy, including labeling methods, ablation strategies, and ablation endpoints, during the CNA procedure. Purely anatomy-guided CNA (15, 16) and intracardiac high-frequency stimulation (HFS)-guided CNA (17) were clinically performed in patients with symptomatic bradycardia caused by excessive vagal tone. The former procedure tends to enlarge the ablation area first and the latter procedure perhaps causes myocardial scarring, leading to other types of arrhythmias. The latter easily causes severe discomfort to the patient during HFS. With regard to the target GPs and chamber during the performance of the CNA procedure, the team of Dr. Pachon and Dr. Aksu tended to ablate all of the atrial GP groups in both atria (4, 8, 16, 17), while Dr. Yao’s team (5) mainly focused on the GP groups in the left atrium, and Dr. Debruyne (18, 19) ablated the GP groups only in the right atrium. The determination of ablation endpoints has not been standardized to date. The techniques of inhibition of the vagal response, elimination of the intracardiac fractionated electrograms, and effecting improvements in electrophysiological parameters, such as increasing the heart rate and shortening the PR interval, have all been used by clinical investigators as ablation endpoints during the performance of the CNA procedure.

In our study, purely anatomy-guided CNA rather than intracardiac HFS–guided CNA was applied because most of the child's rhythms displayed junctional escape rhythms at the onset of the CNA procedure, all of the atrial GP locations in both atria were ablated, and the intracardiac fractionated electrograms in each GP location were used as ideal target intracardiac electrograms. Each ablation target employed 30 W of energy for 30 s with an ablation index of 350–400. Partial return to sinus rhythm and increased heart rate were used as ablation endpoints. The above ablation strategy helped achieve satisfactory clinical outcomes with no perioperative complications.

### Limitations

Despite our obtaining satisfactory clinical results at a 6-month follow-up in our study, the CNA technique is not without its problems and limitations. GPs located in the atrial epicardium, and the medium- and long-term effects (20, 21) of ablation at the endocardial surface, need to be observed before conclusions can be drawn. Meanwhile, a partial reinnervation of the cardiac ganglion after GP ablation in dogs has been reported in the literature (22). It has also been found that the regenerative capacity of nerve fibers is higher in children than in adults, which may be a high-risk factor for producing poor long-term results or recurrence. Hence, multi-center, large-sample clinical studies are needed for the application of CNA in pediatrics.

## Conclusions

In our study, CNA was successfully performed in a child whose rhythm returned to sinus rhythm and clinical symptoms disappeared after 6 months of follow-up. CNA may be an ideal therapeutic strategy for severely symptomatic bradycardic children with appropriate indications. However, the long-term safety and efficacy of CNA in children need to be further evaluated.

## Data Availability

The original contributions presented in the study are included in the article/Supplementary Material, further inquiries can be directed to the corresponding author.
